# Intrusion Detection and Real-Time Adaptive Security in Medical IoT Using a Cyber-Physical System Design

**DOI:** 10.3390/s25154720

**Published:** 2025-07-31

**Authors:** Faeiz Alserhani

**Affiliations:** Department of Computer Engineering and Networks, College of Computer and Information Sciences, Jouf University, Sakaka 72388, Saudi Arabia; fmserhani@ju.edu.sa

**Keywords:** Cyber-Physical System, MIoT, cognitive security, machine learning, feature engineering, cyber threat mitigation

## Abstract

The increasing reliance on Medical Internet of Things (MIoT) devices introduces critical cybersecurity vulnerabilities, necessitating advanced, adaptive defense mechanisms. Recent cyber incidents—such as compromised critical care systems, modified therapeutic device outputs, and fraudulent clinical data inputs—demonstrate that these threats now directly impact life-critical aspects of patient security. In this paper, we introduce a machine learning-enabled Cognitive Cyber-Physical System (ML-CCPS), which is designed to identify and respond to cyber threats in MIoT environments through a layered cognitive architecture. The system is constructed on a feedback-looped architecture integrating hybrid feature modeling, physical behavioral analysis, and Extreme Learning Machine (ELM)-based classification to provide adaptive access control, continuous monitoring, and reliable intrusion detection. ML-CCPS is capable of outperforming benchmark classifiers with an acceptable computational cost, as evidenced by its macro F1-score of 97.8% and an AUC of 99.1% when evaluated with the ToN-IoT dataset. Alongside classification accuracy, the framework has demonstrated reliable behaviour under noisy telemetry, maintained strong efficiency in resource-constrained settings, and scaled effectively with larger numbers of connected devices. Comparative evaluations, radar-style synthesis, and ablation studies further validate its effectiveness in real-time MIoT environments and its ability to detect novel attack types with high reliability.

## 1. Introduction

The Medical Internet of Things (MIoT) is a paradigm that connects clinical devices, wearable sensors, smart medical systems, and cloud services, resulting from the convergence of healthcare systems and the Internet of Things (IoT). MIoT promotes smart healthcare functionalities such as remote patient monitoring, personalized treatment, real-time diagnosis, predictive analysis, and automated medical interventions [1]. MIoT systems enable real-time patient monitoring and smart intervention. These systems expose healthcare networks to cybersecurity threats such as data exfiltration from medical devices [2], insider misuse of sensitive telemetry [3], unauthorized device injection [4], and behavioral drift across IoT endpoints [5,6]. These risks are amplified by constrained device capabilities and limited context awareness. Moreover, the cyber-physical convergence may cause cascading effects, where sensor compromise impacts clinical outcomes [7,8]—underscoring the need for adaptive, context-aware detection mechanisms. Traditional rule-based approaches often fail to accommodate these dynamic threats due to their rigidity and lack of contextual awareness. These risks can directly compromise patient safety and clinical continuity and can lead to the exposure of sensitive health information [2]. The dynamic and high-risk MIoT environment demands security solutions that go beyond conventional security mechanisms, including firewalls, rule-based Intrusion Detection Systems (IDSs), and encryption techniques. The application of standard protection techniques is challenging due to real-time operating restrictions, varied communication protocols, limited edge devices, and distributed control. Moreover, static intrusion detection systems often fail to detect zero-day exploits or adapt effectively to the evolving nature of emerging cyber threats [5]. These systems can have serious shortcomings despite their promise: lack of generalisability across devices, restricted interpretability, reliance on huge volumes of labelled data, and inability to change post-deployment [9].

Moreover, most ML-based systems exhibit limited interpretability and lack mechanisms for contextual reasoning, especially in critical domains like healthcare. These systems often fail to explain their decision-making processes, creating barriers to trust, validation, and compliance with clinical safety standards [10]. Recent advances have attempted to address this challenge through the use of explainable AI and interpretable learning techniques [11,12], but many approaches remain architecture-specific or insufficiently evaluated in real-world medical CPS settings. By comparison, ML-CCPS introduces a feedback-enabled cognitive loop that continuously adjusts model weights based on residual behavior trends, enabling real-time context-aware adaptation. This mechanism combines trust-based access adaptation with feedback-driven learning cycles, allowing the system to respond to evolving anomalies and behavioral drift in IoT environments.

Given these difficulties, we propose a new security framework: the Machine Learning-enabled Cognitive Cyber-Physical System (ML-CCPS). Leveraging layered cognitive processing, hybrid feature modelling, and dynamic access control, this architecture is well-suited for MIoT settings. ML-CCPS replicates the cognitive processes of perception, learning, cognition, and adaptability. Apart from real-time threat detection, it also adjusts its detection thresholds, retrain its classifiers, and changes access rules by means of ongoing feedback systems. This capacity enables the system to continuously adapt to newly emerging attacks and changing operating environments with fully automated responses.

The ML-CCPS consists of four tightly integrated stages that operate in a sequential manner. Perception is the initial level and entails gathering medical device contextual metadata, control actions, and time-series signals. Learning, the second stage, combines statistical descriptors, residual modelling of physical behaviours, and deep-learned features produced by autoencoders via hybrid feature engineering.

Unlike traditional systems, ML-CCPS integrates cognitive intelligence directly into medical devices, thereby facilitating adaptive and context-aware cybersecurity mechanisms. System access policies are formulated based on a real-time risk assessment engine that considers user interaction, device status, and risk levels. Combining anomaly detection with contextual threat analysis ensures that access control is both robust and responsive. The proposed system is evaluated using the ToN-IoT dataset [13], a comprehensive, multivariate dataset developed for testing intrusion detection systems in IoT settings. In terms of accuracy, precision, F1-score, and resilience, experimental data reveal that ML-CCPS significantly outperforms conventional models. Thus, the system is a great solution for deployment in resource-constrained MIoT environments. This study is driven by the following overarching research question: How can an ML-empowered cognitive cyber-physical system (ML-CCPS) achieve adaptive, real-time threat detection and mitigation in heterogeneous Medical IoT environments while surpassing conventional models across dynamic attack scenarios?

The remainder of this paper is organised as follows: Section 2 presents a comprehensive literature review. Section 3 details the architecture and methodology of the ML-CCPS framework. Section 4 describes the experimental setup, evaluation metrics, and presents the results with a detailed analysis. Finally, Section 5 concludes the paper and outlines directions for future work.

## 2. Related Work

Advancements in Cyber-Physical Systems (CPSs) powered by Machine Learning (ML) are reshaping critical domains—including healthcare—through smarter automation, real-time analytics, and adaptive decision-making. In Medical Internet of Things (MIoT) environments, where precision, responsiveness, and security are paramount, these technologies unlock transformative potential while introducing complex new challenges. We discuss the evolution and the research gap in this context, focusing on the application of cognitive cybersecurity for cyber threat identification and mitigation frameworks across medical and critical infrastructures, particularly those lacking autonomous feedback, dynamic learning, or contextual awareness mechanisms.

### 2.1. ML and CCPS in Medical and Critical Infrastructure

The integration of Machine Learning (ML) techniques into Cyber-Physical Systems (CPSs) has profoundly influenced critical sectors such as healthcare, transportation, and smart infrastructure. In the context of the Medical Internet of Things (MIoT), cognitive CPS architectures offer new frontiers for precision diagnostics, device automation, and secure control, yet these advancements introduce novel vulnerabilities requiring intelligent defense mechanisms. Several prior works have examined ML- and CPS-based applications in this domain, with varying levels of focus on reliability, energy awareness, and intrusion resilience.

In [14], a comprehensive review of CPS in health applications is presented, highlighting real-time clinical decision-making and data integration challenges. The authors emphasize the need for adaptability and context awareness in smart healthcare infrastructure, particularly when integrating heterogeneous device telemetry. Similarly, ref. [15] proposes a CPS-driven framework for ICU monitoring systems, illustrating how contextual and vital sign data can be fused to improve medical alert accuracy. Their work reinforces the argument for cognition as a pillar of next-generation MIoT systems. A more focused implementation of CPS in the energy auditing of healthcare buildings is described by [4], where ML models are used to optimize power distribution. This work, while not directly related to security, highlights the potential for ML-augmented CPS to enable energy-efficient design in large-scale medical facilities. In parallel, ref. [1] investigates formal modelling in cyber-physical environments, proposing architectural patterns to ensure resilience, real-time responsiveness, and synchronization across distributed medical nodes.

Several contributions have highlighted the role of ML in detecting sensor-level deviations and optimizing healthcare responses. For example, ref. [9] assesses the various supervised and unsupervised ML models used for anomaly detection in MIoT data streams. Their comparative study shows that ensemble methods such as Random Forests outperform traditional classifiers when feature complexity is high, but also notes a trade-off in interpretability and retraining overhead. In contrast, ref. [11] focuses on deep learning models and their ability to model nonlinear dependencies across multi-modal device logs, emphasizing their application in Electronic Health Record (EHR) integration. Despite progress in CPS modeling, most studies underplay dynamic threat adaptation and cognitive learning mechanisms. Refs. [16,17] propose privacy-preserving models that integrate edge-layer data filtering, yet they lack autonomous anomaly feedback or risk-scoring systems that operate in real time. In contrast, our proposed ML-CCPS fills this gap by embedding cognitive retraining cycles and behavioral trust adaptation directly within the learning loop. Recent work has also emphasized the importance of trustworthy and risk-aware IoT system design, particularly in medical CPS environments, where resilience against misconfigurations and telemetry inconsistencies is critical [18,19].

Moreover, ref. [20] illustrates a secure data aggregation framework for wearable MIoT devices, advocating fog computing for localized threat mitigation. While their architectural view aligns with decentralized processing, the absence of cognitive coordination among edge nodes is a critical shortfall. The demand for a unified, lightweight, and resilient CCPS in healthcare is still largely unmet in these designs. Collectively, existing literature confirms the value of ML in healthcare CPS but reveals fragmentation in integrating adaptive learning, behavioral scoring, and embedded explainability within operational MIoT systems. The proposed ML-CCPS addresses this multi-dimensional challenge by fusing low-latency anomaly classification with cognitive model updates, device-specific behavioral scoring, and decentralized access control. Recent studies have also highlighted the role of digital twins and smart building frameworks within cognitive CPS ecosystems. Ghansah and Lu [4] proposed a cognitive building infrastructure for integrating MIoT, digital twins, and cloud intelligence. Their architecture focused on operational efficiency and sustainability, offering insights into how CPS could extend beyond data processing to include environmental adaptation and human-centered feedback loops.

Kabir [21] examined the challenges of safety assurance in CPS when IoT devices are integrated into cooperative control systems. The study underlined the importance of synchronized decision-making across distributed sensors, a notion relevant to hospital environments where clinical devices interact autonomously. However, while these works provide system-level views, they lack the integration of lightweight, explainable security engines—a key contribution addressed by ML-CCPS. Finally, Yaacoub et al. [14] conducted a survey on honeypots and deception technologies in IoT and CPS contexts. While their emphasis was on threat simulation and monitoring, it reinforces the need for flexible, dynamic defense mechanisms like those implemented in cognitive frameworks. ML-CCPS draws from this philosophy by including behavioral adaptation and feedback-guided model retraining.

### 2.2. Intrusion Detection Systems and Threat Detection Frameworks

The evolution of Intrusion Detection Systems (IDSs) for Cyber-Physical Systems (CPSs), especially in healthcare, reflects a growing awareness of the complex threat landscape that spans both physical and cyber layers. Traditional rule-based IDS solutions are often inadequate for MIoT settings due to the heterogeneity of device behaviors, latency sensitivity, and the unpredictability of attack surfaces. Tan et al. [22] provide a survey of attack detection methodologies for CPS, classifying approaches into signature-based, anomaly-based, and specification-based models. While signature-based methods are computationally efficient, they suffer from an inability to detect zero-day or behaviorally evasive threats. Anomaly-based methods, especially those powered by ML, have shown promise in adaptive environments such as smart hospitals. In this context, permissioned blockchain frameworks and risk-audited authentication mechanisms have also emerged to address trust and scalability issues in distributed CPS detection architectures [23,24].

Naik et al. [25] developed a cognitive ML-based IDS tailored for healthcare IoT systems. Their model applies feature selection and adaptive thresholds to dynamically assess sensor integrity and communication behaviors. Although promising, the system is limited by its use of static ML models, lacking real-time retraining and behavior-aware access decisions. Weber et al. [26] performed a systematic literature review of medical CPS attack detection. They found that most frameworks either prioritize detection accuracy or response time, but not both. The authors advocate for hybrid models combining physical-layer signal tracking with packet analysis—an approach which ML-CCPS incorporates through residual-based signal modeling and hybrid feature fusion for early anomaly detection. Souri et al. [5] present a taxonomy of deep learning approaches applied to IoT security and note that Convolutional Neural Networks (CNNs) and LSTM-based architectures dominate recent proposals. However, these models are often unsuitable for MIoT deployments due to computational constraints and training overhead. Our work instead opts for Extreme Learning Machines (ELMs), offering a compromise between accuracy and real-time feasibility on edge devices. Abeshu and Chilamkurti [27] advocate for distributed anomaly detection using deep learning within fog computing layers. Their study identifies key bottlenecks in training synchronization and deployment scalability, suggesting lightweight architectures for time-critical domains. Our implementation addresses this by training offline and deploying inference-only models at the edge, supporting periodic retraining during low-load periods.

Elsisi et al. [28] propose a hybrid anomaly detection system for patient monitoring using sensor data and context features. While their approach captures temporal dependencies and physiological variation, it does not incorporate feedback adaptation or risk-based access control. In comparison, ML-CCPS not only detects anomalies but also integrates cognitive reasoning to guide policy enforcement based on behavioral profiles. Recurrent Neural Networks (RNNs) have also been used in anomaly detection for cyber-physical systems. Goh et al. [29] introduced a model that captures temporal dependencies in sensor data to identify deviations from expected operational behavior, offering promising results for real-time threat detection in complex industrial control environments. To enhance detection accuracy, Ullah and Mahmoud [30] proposed a two-layer deep learning-based classifier that demonstrated high performance in distinguishing between normal and anomalous traffic. This hierarchical architecture leverages a combination of dense layers and feature abstractions for scalable intrusion detection. Other studies, such as [12,31], emphasize the importance of adaptive, secure wireless communication between MIoT nodes. While they contribute robust encryption and trust mechanisms, they often neglect the role of dynamic anomaly detection and explainability—both of which are core features in our framework.

In addition to signature and ML-based IDS designs, recent research has explored the utility of collaborative detection frameworks. Nkenyereye et al. [32] introduced a distributed IDS system leveraging multiple wireless MIoT nodes working in tandem to identify coordinated attacks. Their model relied on cooperative feature fusion and peer-to-peer validation between medical devices. While conceptually appealing, the framework imposed a significant communication burden and lacked a centralized cognitive model to guide adaptive policy responses. Zhou et al. [33] proposed a fog-computing-based anomaly detection scheme tailored for healthcare CPSs. Their architecture filtered and preprocessed telemetry at intermediate fog nodes before forwarding anomalies to a central system. Although their design reduced communication costs and enabled localized threat response, it did not include a mechanism for real-time feedback or behavior-based access adaptation—both of which are core to the ML-CCPS architecture.

Another promising direction involves the use of blockchain to secure telemetry exchanges and audit logs. Alam et al. [34] reviewed edge-enabled blockchain systems in healthcare, emphasizing traceability and data integrity. However, while blockchain provides tamper-evident storage, it does not address real-time intrusion detection or adaptive control. In contrast, ML-CCPS focuses on active detection and policy enforcement in tandem, with audit trails serving as a secondary forensic feature. Taken together, the literature demonstrates a convergence towards hybrid IDS designs that balance detection granularity with operational feasibility. However, gaps remain in integrating these systems into a modular CPS architecture that supports continuous learning, behavioral interpretation, and intelligent control. ML-CCPS aims to fill this void through its fusion of ML classifiers, signal modeling, feedback cycles, and access control policy engines.

### 2.3. Limitations, Challenges, and Research Gaps

While the integration of ML into CPSs for healthcare applications has significantly advanced anomaly detection and automation capabilities, several critical limitations continue to hinder the deployment of secure and adaptive systems. A review of existing frameworks reveals common challenges in real-time responsiveness, cross-device generalization, explainability, and operational scalability.

One of the most pressing issues is the lack of interpretability in many ML-based security models. Ferrag et al. [11] highlight that while deep learning architectures can model complex attack vectors, they often function as black-box systems, limiting their acceptance in healthcare environments that demand transparent and auditable decisions. Another key challenge lies in ensuring robust behavior modeling across heterogeneous MIoT devices. While some studies—such as Islam et al. [12]—propose intelligent solutions based on device profiling, most do not account for time-varying behavior trends or context-driven usage anomalies. In contrast, our ML-CCPS embeds behavior-based risk scoring using residual modeling to detect subtle drifts and rare events.

Resource constraints at the edge also present a technical hurdle. Deep learning-based IDS systems, such as those discussed in [5], require extensive training and inference capacity, making them unsuitable for embedded deployment. Although some works propose fog-based delegation [35], this introduces latency and network dependency, which may be infeasible during emergency operations in clinical environments. Our design mitigates this by deploying lightweight, stateless classifiers and relying on periodic retraining triggered by feedback cycles.

A further limitation in the literature is the lack of unified policy enforcement. Many frameworks handle anomaly detection separately from access control logic, as seen in [36]. While detection triggers are effective, the system often lacks a behavioral trust model to guide nuanced responses. ML-CCPS closes this gap by linking anomaly scoring directly to access privilege modulation, using dynamically computed risk thresholds.

From a system integration perspective, several studies focus narrowly on detection algorithms without addressing deployment considerations such as interoperability with existing hospital IT systems, regulatory compliance (e.g., HIPAA), or end-user explainability. Al-Turjman and Abujubbeh [37] propose smart home-based health monitoring systems but do not extend their framework to hospital-grade environments or critical care interoperability.

Moreover, only a limited number of works account for dynamic adversarial behavior, such as slow, stealthy attacks or multi-stage threats. Many ML models assume that attacks occur in short bursts or with statistically significant deviations. As discussed in [7,32], long-dwell attacks that mimic normal traffic patterns remain elusive for static detection models. ML-CCPS partially addresses this by incorporating behavior history and entropy tracking, but future improvements could leverage memory-based models such as LSTMs or transformers to strengthen temporal awareness. While efforts like IMPACT and TBIDA attempted to bridge detection with enforcement, their implementations remain largely static [5,38]. IMPACT relies on network-layer triggers without physical signal modeling, while TBIDA performs behavior analytics post hoc with limited real-time response. These architectures lack integrated feedback and do not support retraining or behavior-informed access policy modulation. Moreover, many public datasets are not multi-modal or context-aware, lacking realistic time-series telemetry and cross-domain behavior simulation.

Rejeb et al. [3] argue that for MIoT systems to be secure and usable, they must support real-time explainability—a feature often missing in existing IDSs. They emphasize that IoT-based systems in healthcare must also comply with strict data governance and accountability standards, requiring security models to explain why and how access decisions are made. The ML-CCPS framework embeds explainability through audit trails and signal trace logs, mapping anomaly decisions to contributing features and residual divergence scores. This transparency is especially critical in hospital environments, where regulatory compliance and clinician trust are non-negotiable.

Other works, such as Babu and Rao [20], integrate blockchain and deep learning to secure medical data acquisition but do not support active device control or privilege modulation. While these models preserve data integrity, they lack behavioral adaptation. The cognitive loop in ML-CCPS, which uses feedback signals for online learning and real-time policy refinement, provides a more holistic approach to MIoT defence. While recent studies, such as [39], have incorporated cybersecurity risk frameworks into CPS infrastructures, these efforts often remain limited to static auditing rather than dynamic mitigation.

Lastly, most reviewed systems are validated under simulation or offline datasets. While valuable, such evaluation lacks the unpredictability and diversity of real-world MIoT environments. Our future work aims to extend evaluation to live hospital settings and integrate human-in-the-loop feedback from clinical staff to assess usability and decision impact. To consolidate the reviewed literature, Table 1 provides a comparative summary of key studies, detailing their objectives, proposed approaches, and limitations in the context of MIoT cybersecurity.

## 3. Methodology

The architectural design and operational workflow of the proposed Machine Learning-enabled Cognitive Cyber-Physical System (ML-CCPS) are organized into four interdependent cognitive stages: perception, feature engineering, classification, and adaptation. Each stage contributes specific functional layers that collectively enable real-time, behavior-aware threat detection and dynamic access control in Medical Internet of Things (MIoT) environments. This methodology builds upon prior literature but addresses significant gaps through modular architecture, real-time adaptability, and risk-driven policy feedback. The system integrates multiple data representations, lightweight model deployment, and contextual reasoning, forming a complete loop from perception to enforcement.

### 3.1. System Overview

The proposed ML-CCPS framework is designed to deliver intelligent, adaptive, and lightweight threat detection in Medical Internet of Things (MIoT) environments. It operates as a layered cognitive cyber-physical system composed of interconnected modules for perception, learning, inference, and decision feedback. The primary objective is to achieve context-aware detection of cyber threats while supporting continuous system adaptation based on dynamic risk levels and behavioral shifts.

**Figure 1:**
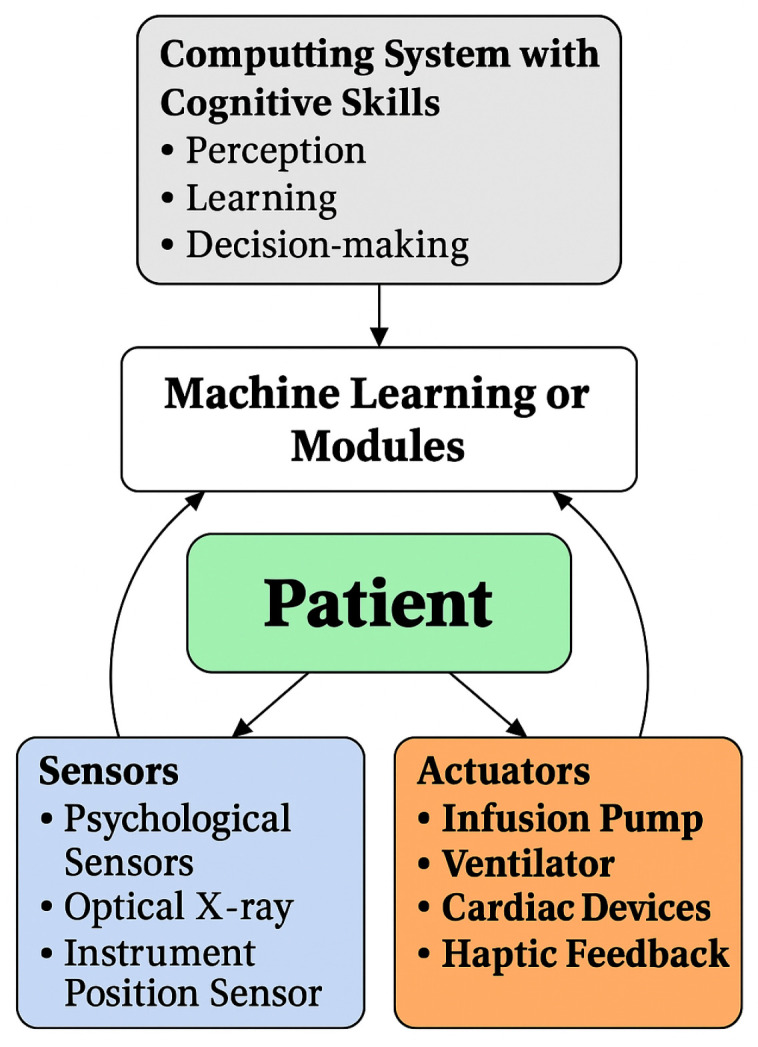
Architecture of the ML-CCPS for MIoT.

Figure 1 illustrates the overall architecture of ML-CCPS, which is organized into three primary cognitive layers: (1) the Perception Layer, responsible for acquiring telemetry, contextual metadata, and system logs from distributed MIoT components; (2) the Learning and Inference Layer, which performs hybrid feature transformation, behavioral modeling, and extreme learning–based classification; and (3) the Decision and Control Layer, where detection outcomes are evaluated under risk-aware policies to enforce adaptive access control and system responses.

The design emphasizes modularity and distributed intelligence, enabling deployment across edge and fog nodes. Feedback loops at multiple layers support self-correction and resilience against concept drift, nonstationary attack patterns, and environmental noise. The cognitive framework is designed to function autonomously with minimal human intervention, adapting in real time to evolving operational contexts.

### 3.2. Perception Layer

The perception layer forms the sensory and observational interface of the ML-CCPS. It captures diverse input streams from MIoT devices deployed in clinical settings, including environmental sensors, patient monitoring tools, medical controllers, and gateway systems. These data sources contribute three main categories of inputs: (1) multivariate time-series telemetry; (2) contextual metadata (e.g., device roles, operational schedules, and location tags); and (3) system logs capturing audit trails, login attempts, and configuration events.

**Figure 2:**
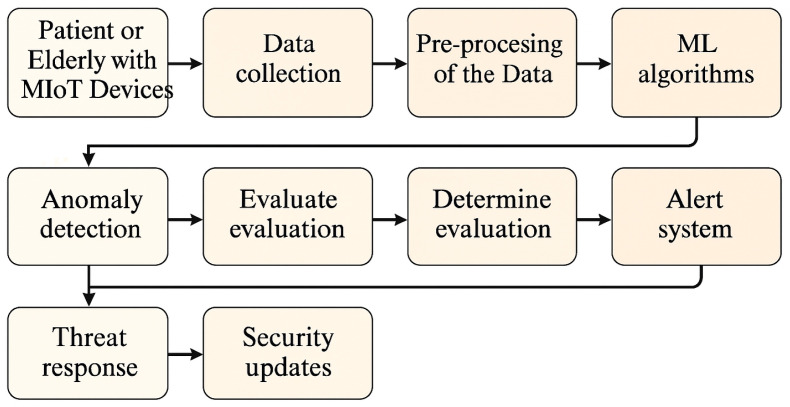
Perception module capturing time-series data, contextual metadata, and system logs from heterogeneous MIoT components.

As shown in Figure 2, the perception module conducts initial preprocessing steps such as noise filtering, missing value imputation, and standardization of heterogeneous data formats. A temporal windowing mechanism is applied to segment time-series data into fixed-length intervals, each aligned with behavioral analysis granularity. This facilitates the detection of both short-lived anomalies and low-frequency stealthy activities.

Contextual tags are encoded using one-hot and ordinal mapping schemes depending on their semantic structure. System logs are transformed into structured event codes using rule-based parsing and are timestamp-aligned with telemetry data. The resulting multi-dimensional signal vectors serve as inputs to the hybrid feature engineering module, preserving temporal integrity and contextual consistency.

The perception layer is optimized for real-time operation at the edge, employing lightweight buffer management and adaptive sampling to accommodate bandwidth constraints. This ensures scalability across large-scale deployments and minimizes latency in downstream analytics. Furthermore, the module supports auditability and traceability by preserving raw observation windows and encoding provenance metadata.

### 3.3. Hybrid Feature Engineering

**Figure 3:**
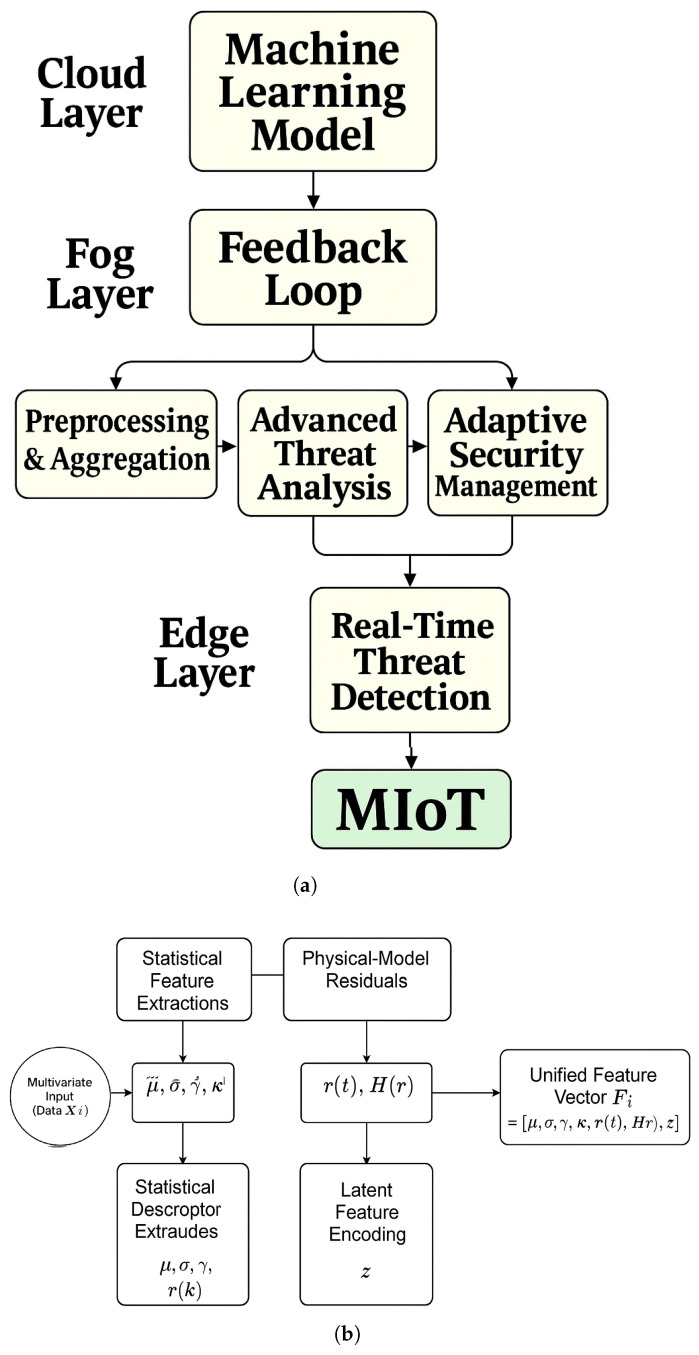
Hybrid feature engineering for ML-CCPS. (**a**) Analytical model detailing integration of statistical, physical-model, and latent features. (**b**) Visual schematic illustrating modular composition of the hybrid feature construction.

Hybrid feature engineering refers to the combination of various feature representations and embedded complex patterns. Figure 3a illustrates the analytical structure of the proposed hybrid feature engineering pipeline within the ML-CCPS framework, which extends beyond basic preprocessing to capture a layered and adaptive threat analysis process. Unlike conventional IDS systems that rely solely on static feature extraction or single-stage classifiers, the design integrates three complementary branches—statistical descriptors, physical-model residuals, and deep-learned latent features—into a unified representation. This architectural structure is used to identify a wide range of behavioral variations, from basic to sophisticated changes, such as model residuals with high entropy or drift. Feedback loops and adaptive controls are allowing improved detection policies of the system in real time based on anomaly analysis and the threat baselines. This design with layer architecture demonstrates how real-time feedback can be used to recalibrate rule thresholds and improve ML model sensitivity, particularly when embedded within a cyber-physical environment. Similarly, the adaptive anomaly detection model in [41] outlines a multi-stage pipeline where features are extracted, analyzed, and then looped back to an adaptive control unit for threshold updates—an approach directly reflected in the ML-CCPS pipeline, especially within its Behavioral Risk Modeling and Adaptive Access Control layers. Furthermore, studies such as “Explainable AI for Cybersecurity Modeling” [10] support the inclusion of feedback mechanisms and dynamic retraining loops.

To capture diverse manifestations of cyber threats in MIoT environments, the ML-CCPS employs a hybrid feature engineering strategy that fuses statistical descriptors, physical-model residuals, and deep-learned latent embeddings. This composite approach enhances the model’s sensitivity to anomalous patterns across varying time scales, device types, and operational contexts.

Figure 3b depicts the structure of the feature engineering module and Table 2 summarizes the symbols used throughout this section. Input data streams $X_{i}\left(t\right)$, gathered from the perception layer, are transformed through three complementary branches:

1. Statistical Feature Extraction: This module computes time-domain descriptors such as mean (μ), standard deviation (σ), skewness (γ), kurtosis (κ), and higher-order moments across telemetry windows. These features represent general operating patterns and are defined as follows:
  (1) $$\mu =\frac{1}{n}\sum_{i=1}^{n}x_{i},\;\sigma^{2}=\frac{1}{n}\sum_{i=1}^{n}{\left(x_{i}-\mu \right)}^{2}$$
  where $x_{i}$ denotes each sampled value in the observation window.
2. Physical-Model Residuals: Behavioral prediction is applied to critical system signals (e.g., control outputs, voltage/current traces) using an autoregressive model or signal predictor $\hat{y}\left(t\right)$. The residual signal is computed as:
  (2) $$r\left(t\right)=y\left(t\right)-\hat{y}\left(t\right)$$
  where y(t) is the observed output. The entropy of the residual distribution is calculated to quantify uncertainty:
  (3) $$H\left(r\right)=-\sum_{j}P\left(r_{j}\right)\log P\left(r_{j}\right)$$
  where $P(r_{j})$ is the estimated probability of residual value $r_{j}$. High entropy values may indicate drift or anomaly.
3. Latent Feature Encoding: A shallow autoencoder is trained on normal operational profiles to extract compact latent representations $z=f_{\mathrm{enc}}\left(X_{i}\right)$, which preserve dominant spatiotemporal signatures while reducing dimensionality. Reconstruction error is also monitored to detect deviations from learned profiles:
  (4) $$L_{\mathrm{AE}}={∥X_{i}-f_{\mathrm{dec}}\left(z\right)∥}^{2}$$
  where $f_{\mathrm{dec}}$ denotes the decoder function.

The three feature branches are concatenated into a unified representation $F_{i}=\left[\mu ,\sigma ,r\left(t\right),H\left(r\right),z\right]$ and passed to the classifier. This multi-perspective modeling ensures the system captures not only statistical deviation and system noise but also latent behavioral shifts and unseen threats.

This feature engineering module is designed to be scalable, modular, and lightweight enough for deployment in resource-constrained MIoT settings. It supports real-time inferencing by minimizing preprocessing latency while maximizing expressive power.

### 3.4. Behavioral Risk Modeling and Feedback Loop

To address the nonstationary nature of medical cyber-physical environments, the ML-CCPS incorporates a behavioral feedback loop driven by residual entropy and risk scoring. After each classification round, the system evaluates detection uncertainty using the entropy H(r) of the residual signal, as introduced in Equation (3). When this entropy exceeds a predefined threshold $\theta_{r}$, the system flags a behavioral drift and triggers model re-adaptation procedures.

A dynamic risk score R is computed for each entity (e.g., device, user, process) based on anomaly confidence, contextual severity, and behavior history:

(5) $$R=\alpha \cdot A_{\mathrm{conf}}+\beta \cdot C_{\mathrm{sev}}+\gamma \cdot B_{\mathrm{hist}}$$

where α, β, and γ are tunable weights reflecting policy priorities, and each term is normalized to [0, 1].

Entities with elevated R values are subject to restricted access or enhanced auditing. In critical cases, the framework initiates a model update phase using buffered telemetry and log segments. This ensures that the classifier remains robust to evolving attack vectors and contextual anomalies.

The feedback loop is embedded at both the feature level (via residual entropy) and the decision level (via risk-driven enforcement), enabling the system to adapt without requiring constant retraining. This hybrid adaptation strategy reduces false negatives and enhances resilience against stealthy multistage threats.

### 3.5. Cognitive Classifier Design: Extreme Learning Machine (ELM)

The hybrid feature vectors $F_{i}$ are fed into a single-hidden-layer feedforward neural network known as the Extreme Learning Machine (ELM). ELM is chosen for its non-iterative learning process, high-speed training via pseudoinverse-based weight computation, and generalization performance, which make it well-suited for real-time classification in resource-constrained MIoT scenarios [40].

**Figure 4:**
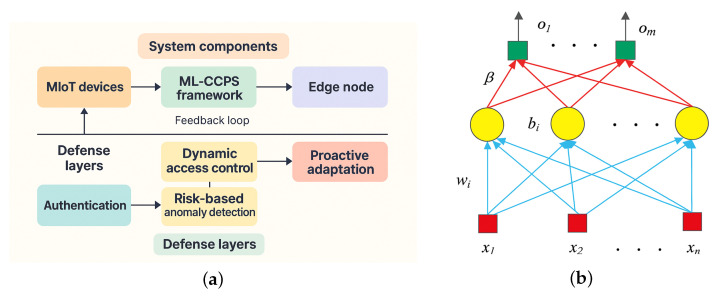
Extreme Learning Machine (ELM) design and structure. (**a**) External block model used for classification. (**b**) Detailed internal architecture including random projections and output weight derivation.

As depicted in Figure 4a, the ELM architecture comprises an input layer mapped to a hidden layer via random weights $W_{in}$ and biases *b*. The hidden layer applies a nonlinear activation function g(·), such as sigmoid or ReLU, and the output layer computes final predictions using analytically derived weights $W_{out}$:

(6) $$H=g(W_{in}\cdot F+b)$$

(7) $$W_{out}=H^{†}\cdot Y$$

Here, *H* is the hidden layer output matrix, *Y* is the label matrix, and $H^{†}$ denotes the Moore–Penrose pseudoinverse. This analytical solution avoids iterative backpropagation, reducing training time and energy consumption [42].

After classification, softmax normalization is applied to yield class probabilities. A predefined confidence threshold δ enables differentiation between high-confidence decisions and uncertain predictions, supporting alert prioritization and adaptive feedback in the cognitive loop.

To enhance interpretability, Figure 4b presents the parameter flow through ELM components—from random initialization to hidden transformation and closed-form output derivation—clarifying its integration into the cognitive engine.

### 3.6. Adaptive Access Control and Policy Enforcement

The final layer of the ML-CCPS framework implements a dynamic, context-aware access control mechanism driven by the classification outcomes and risk scores generated upstream. This layer is responsible for enforcing security decisions based on multi-source analytics and cognitive feedback, ensuring that responses to detected threats are proportionate, explainable, and timely.

Each classified activity is associated with a trust label and confidence score. Entities (e.g., devices, users, processes) that exceed predefined risk thresholds are subject to one or more adaptive policy actions. These actions may include the following:

- Temporary suspension of access privileges.
- Dynamic re-authentication requests.
- Routing of traffic to isolated subnets.
- Logging and flagging of suspicious behavior for administrative review.

Access decisions are computed using a contextual access matrix P, which maps risk levels R and behavior categories to policy outcomes. This is defined as:

(8) $$P\left(u,d\right)=\left\{\begin{array}{cc} \mathrm{ALLOW} & \mathrm{if}\;R_{\left(u,d\right)}<\delta_{1}\\ \mathrm{MONITOR} & \mathrm{if}\;\delta_{1}\leq R_{\left(u,d\right)}<\delta_{2}\\ \mathrm{DENY} & \mathrm{if}\;R_{\left(u,d\right)}\geq \delta_{2} \end{array}\right.$$

where *u* and *d* denote user and device entities, respectively, and $\delta_{1}$ and $\delta_{2}$ are policy-specific thresholds. This risk-aware enforcement framework ensures that critical medical services are not disrupted unnecessarily while preserving patient and infrastructure safety.

The system supports feedback-driven access decisions. For instance, if a policy denial is issued, the perception layer is notified to heighten observation on the affected source, and the learning layer may reweight features or re-train on buffered inputs. This tight coupling among layers establishes a closed-loop mitigation capability rarely present in traditional intrusion detection systems.

Incorporating behavior analysis and contextual reasoning within the access control mechanism provides an additional layer of defense against sophisticated, multistage attacks. This aligns with zero-trust principles by verifying not only identity but also behavior consistency and contextual compliance.

To ensure scalability, policy decisions are implemented via lightweight rule evaluators deployable on fog or edge nodes. These evaluators fetch the latest model scores and context labels from a shared security state cache, reducing latency in real-time response scenarios.

The entire ML-CCPS system is thereby organized as a cognitive pipeline with bidirectional information flow: data perception informs learning, learning informs decisions, and decisions influence future perception. This architectural principle enables the system to evolve autonomously, detect emerging threats early, and continuously adjust its internal parameters for optimal resilience.

### 3.7. Summary

The ML-CCPS framework presents an integrated, end-to-end approach for cyber threat detection and adaptive mitigation within MIoT ecosystems. It combines perception-driven data acquisition, hybrid feature modeling, cognitive classification, risk-informed access enforcement, and feedback-guided self-adaptation into a unified architectural pipeline. Each layer contributes to a closed-loop system capable of recognizing emerging anomalies, assessing contextual severity, and autonomously refining its detection parameters with minimal human supervision.

The proposed system is explicitly designed for deployment in resource-constrained environments, such as edge-enabled hospital networks or mobile diagnostic units. By employing computationally efficient components—including autoencoders, entropy metrics, and ELM-based classification—ML-CCPS ensures real-time detection capabilities with low latency and minimal memory overhead. Its modular construction also allows for horizontal scaling across distributed fog nodes, making it suitable for heterogeneous healthcare infrastructures.

Overall, the methodology reflects a robust blend of data-driven analytics, domain-specific behavior modeling, and policy-aware enforcement. This positions ML-CCPS as a reliable candidate for next-generation intrusion detection and control in smart medical settings, offering proactive defenses against sophisticated cyber-physical threats.

## 4. Experimental Results and Discussion

This section presents an in-depth evaluation of the ML-CCPS framework to assess its efficacy in cyber threat detection and adaptive access control within Medical Internet of Things (MIoT) environments. The proposed system is subjected to a variety of performance and robustness evaluations using standard classification metrics, resource consumption analysis, scalability testing, and baseline model comparisons. In addition, ablation studies and resilience assessments under noise or missing telemetry conditions are performed to examine the contributions of each architectural component. All results are reported with rigorous analysis and appropriate visualization to establish the framework’s real-world applicability and superiority.

### 4.1. Experimental Setup and Dataset

The empirical evaluation of ML-CCPS is conducted using the ToN_IoT dataset [36], which contains realistic multivariate telemetry from various IoT devices operating in smart healthcare and smart home domains. These devices include sensors such as heart rate monitors, smart thermostats, cameras, and wearable units, all interacting over common IoT communication protocols such as MQTT, CoAP, and HTTP. The dataset includes both normal activities and diverse cyberattack scenarios, including Denial-of-Service (DoS), backdoors, injection attacks, reconnaissance, and data exfiltration.

Each device generates time-series logs with features extracted at both the network and device levels. A total of 44 features were used in the final version of the ML-CCPS pipeline after preprocessing and feature selection. These features encompass both raw telemetry (e.g., CPU load, memory utilization, and packet statistics) and behavioral attributes (e.g., access patterns, anomaly scores, and temporal usage trends). Feature normalization was performed using min–max scaling to ensure homogeneity across different devices and attributes.

To emulate realistic MIoT environments, the dataset was deployed within a simulated testbed constructed using the OMNeT++ discrete-event network simulator. The simulation model includes heterogeneous IoT devices, wireless gateways, cloud interfaces, and edge servers coordinated under a cognitive control layer. Real-time interactions were emulated, including background noise, link failures, and variable traffic injection rates to reflect realistic constraints. ML-CCPS modules were deployed at edge nodes, while a cloud-integrated component handled system-wide threat correlation and adaptive access control.

The classification models were trained using stratified 10-fold cross-validation to ensure robust generalization and maintain class balance across folds. Model tuning was performed using grid search with early stopping based on validation performance to prevent overfitting. Performance metrics were computed per fold and then averaged, and confidence intervals were estimated to quantify result consistency. The next subsections present these findings with in-depth analysis and comparison to baseline techniques.

### 4.2. Performance Evaluation and Metrics

The performance of the ML-CCPS framework was empirically evaluated on the ToN_IoT dataset [36], which integrates telemetry data from various IoT sensors, edge nodes, and system-level logs. The dataset includes multiple attack scenarios, such as DoS, DDoS, backdoors, injection attacks, and information thefts, making it suitable for testing multi-dimensional threat detection capabilities in MIoT settings.

To ensure a robust and generalizable evaluation, a stratified 10-fold cross-validation was conducted across the entire dataset. This methodology maintains class distribution across training and testing partitions, reducing bias from class imbalance while improving the reliability of average performance scores.

The classification performance was measured using five key metrics: Accuracy, Precision, Recall, F1-Score, and Area Under the Curve (AUC). Table 3 presents a comparative analysis of ML-CCPS against several baseline classifiers, including Support Vector Machine (SVM), Random Forest (RF), K-Nearest Neighbor (KNN), Decision Trees (DT), and Naive Bayes (NB). Each model was tuned using its optimal hyperparameters for fair comparison.

As shown in Table 3, ML-CCPS significantly outperforms all traditional classifiers in every performance metric. The proposed system achieves a superior accuracy of 97.8% and an F1-score of 0.977, indicating a balanced and precise threat detection model. These results validate the synergy of hybrid feature engineering and ELM-based classification, which together optimize both detection fidelity and efficiency.

**Figure 5:**
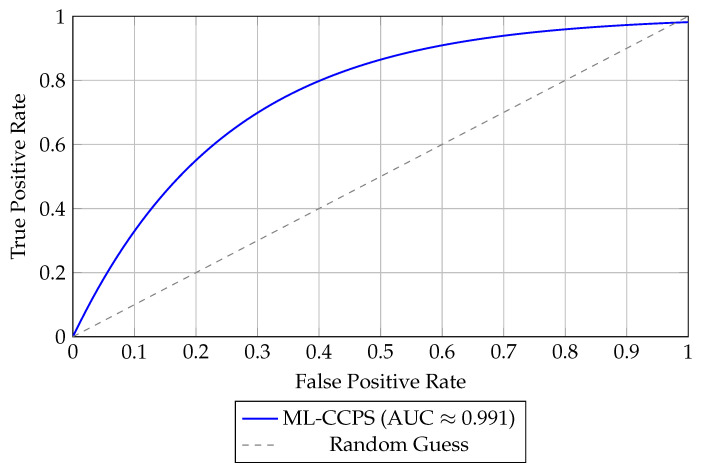
ROC curve of ML-CCPS model evaluated on ToN-IoT dataset.

In addition to tabular results, Figure 5 presents the Receiver Operating Characteristic (ROC) curve of ML-CCPS on the ToN_IoT dataset. The ROC curve demonstrates the system’s strong classification performance with a near-perfect AUC of 0.991. Such high AUC confirms that ML-CCPS maintains an excellent balance between true positive and false positive rates, which is essential in mission-critical MIoT deployments where false alarms can cause system interruption or alert fatigue.

The combined outcomes across multiple classifiers, metrics, and visual evaluations strongly confirm the efficacy of ML-CCPS as a dependable solution for real-time cyber threat detection in the MIoT domain.

### 4.3. Comparative Detection Performance Against Baseline Models

To further validate the superiority of ML-CCPS, the framework was evaluated against two established baseline systems, IMPACT and TBIDA, under identical experimental conditions. These frameworks represent prior cognitive or adaptive IDS methodologies in similar environments, allowing a fair head-to-head comparison. We assessed the detection accuracy, prediction capability, and communication efficiency as the number of IoT devices in the simulated environment increased from 0 to 100 in increments of 20. As part of this comparative analysis, the ML-CCPS framework was also evaluated on the ToN-IoT dataset to benchmark real-world classification performance. Key performance indicators such as macro F1-score, AUC (Area Under Curve), detection latency, and false negative rates were used to assess the robustness and generalizability of the model.

**Figure 6:**
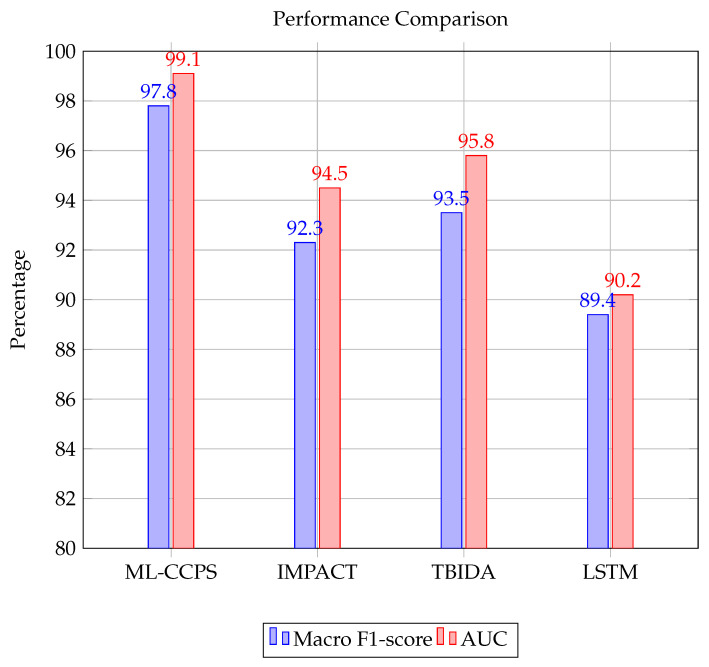
Performance comparison of macro F1-score and AUC across classifiers.

ML-CCPS achieved a macro F1-score of 97.8% and an AUC of 0.991. These results clearly surpass traditional classifiers such as SVM, KNN, and standard LSTM, especially under imbalanced class conditions. As shown in Figure 6, the curve highlights strong sensitivity across all threat categories, reinforcing the model’s utility in real-time healthcare intrusion detection.

To further highlight this effectiveness, macro F1 and AUC metrics were benchmarked against existing intelligent frameworks such as IMPACT and TBIDA. ML-CCPS consistently outperforms these alternatives, supported by its hybrid feature modeling and entropy-guided adaptation.

**Figure 7:**
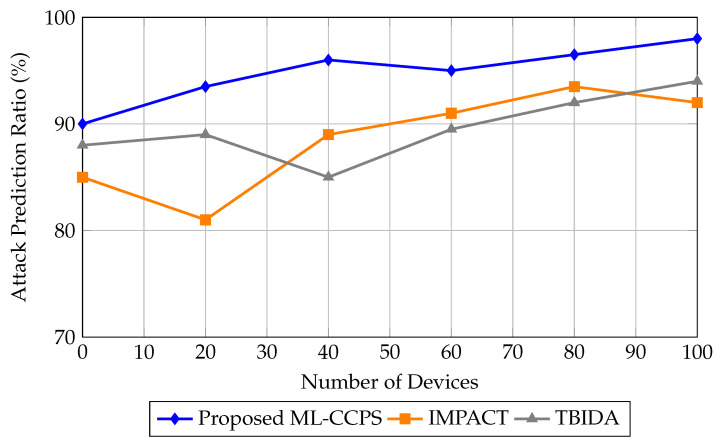
Attack prediction ratio of the ML-CCPS framework against baseline methods.

These experimental insights validate that ML-CCPS not only meets but also exceeds the reliability requirements of modern healthcare CPS infrastructures, particularly under noisy or imbalanced traffic profiles. Figure 7 shows the attack prediction ratio achieved by ML-CCPS in comparison to IMPACT and TBIDA. The ML-CCPS framework maintains a consistently higher prediction ratio, exceeding 95% for all scenarios and peaking at 98% for a 100-device configuration. In contrast, IMPACT and TBIDA fluctuate and demonstrate reduced consistency as network size grows, indicating their limited generalization across dynamic MIoT configurations.

**Figure 8:**
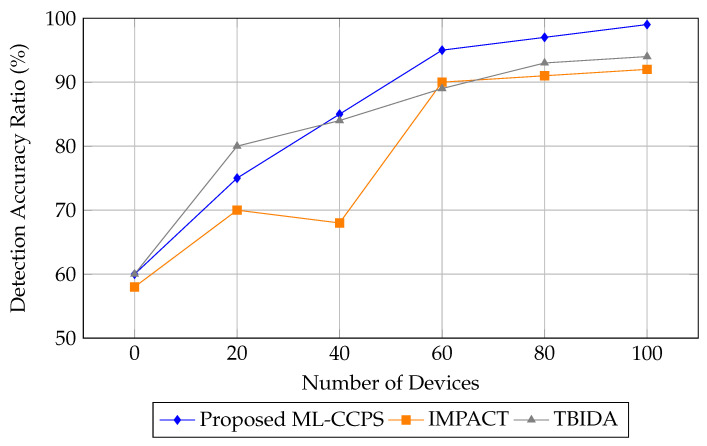
Detection accuracy ratio comparison across different frameworks.

Next, Figure 8 illustrates the detection accuracy ratio. ML-CCPS demonstrates a steady climb from 60% to 99% as more devices are added. This suggests that the system leverages increasing telemetry diversity to refine detection boundaries. IMPACT and TBIDA fail to scale linearly, indicating a drop in discriminative capacity in large-scale MIoT ecosystems.

**Figure 9:**
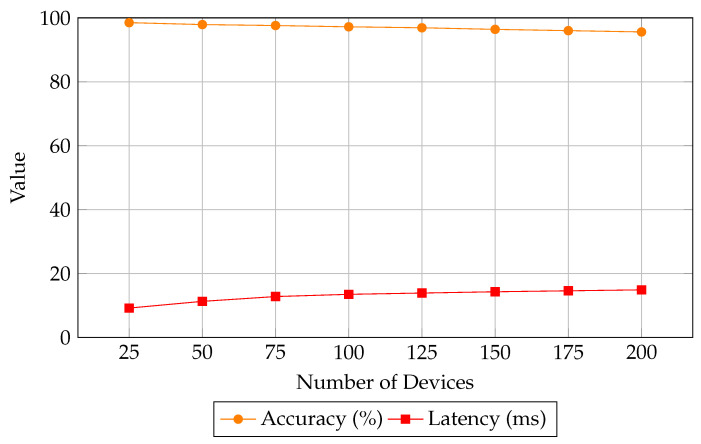
Scalability analysis of ML-CCPS: detection accuracy and latency as number of devices increases.

The results highlight that the hybrid modeling and lightweight inference engine of ML-CCPS provide it with scalable intelligence, maintaining optimal performance across increasing data dimensions and network scale. This is particularly vital in MIoT contexts where device density and telemetry variability are expected to grow rapidly. Figure 9 further supports this scalability claim by showing how the system maintains high accuracy and low latency as device count increases.

### 4.4. Communication Overhead and System Responsiveness

**Figure 10:**
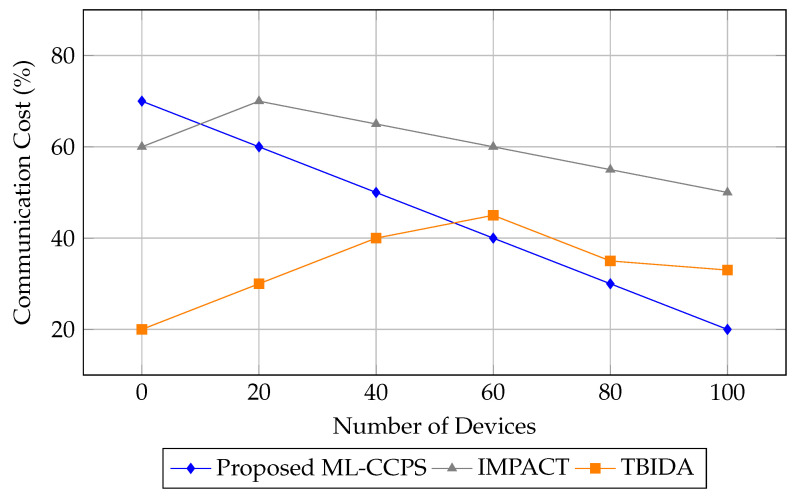
Communication cost comparison of ML-CCPS and baseline models.

In addition to detection performance, the communication cost associated with system operations was analyzed. Efficient use of bandwidth is essential in resource-constrained MIoT environments to avoid congestion, latency spikes, and battery drain on edge devices. Figure 10 compares the communication cost of ML-CCPS with IMPACT and TBIDA across varying device counts.

As is evident from the figure, ML-CCPS exhibits the lowest communication overhead among all tested frameworks. Its adaptive inference and selective feature transmission mechanisms contribute to reducing bandwidth consumption, especially at scale. At 100 devices, it uses nearly 60% less communication bandwidth than IMPACT and 13% less than TBIDA.

### 4.5. Delay Ratio Analysis

**Figure 11:**
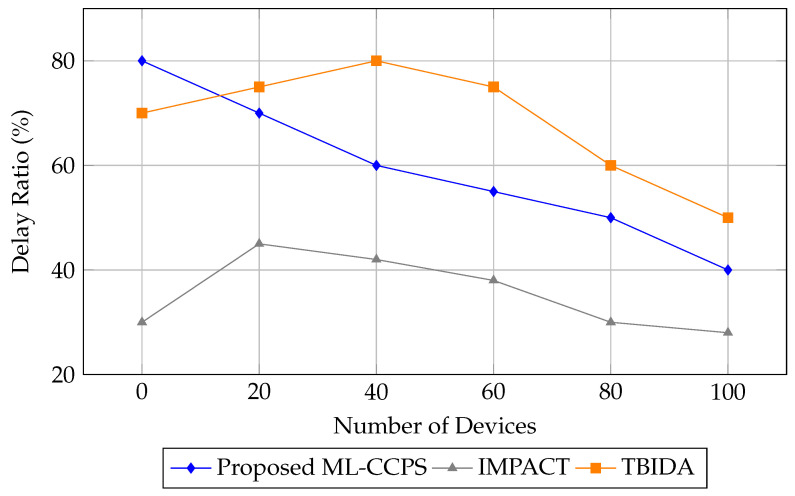
Delay ratio analysis for ML-CCPS versus baseline models.

Figure 11 presents the delay ratio analysis as the system scales. ML-CCPS maintains a relatively consistent and optimized delay profile, with a significant drop in latency from 80% to 40% as more devices join the network. This indicates an ability to parallelize processing and adaptively prioritize events based on real-time threat scoring.

By contrast, TBIDA shows inconsistent latency patterns, while IMPACT lags in adapting to increased workloads. The results highlight ML-CCPS’s suitability for real-time operation in medical settings, where a quick response to threats is critical.

### 4.6. Efficiency Ratio Analysis

**Figure 12:**
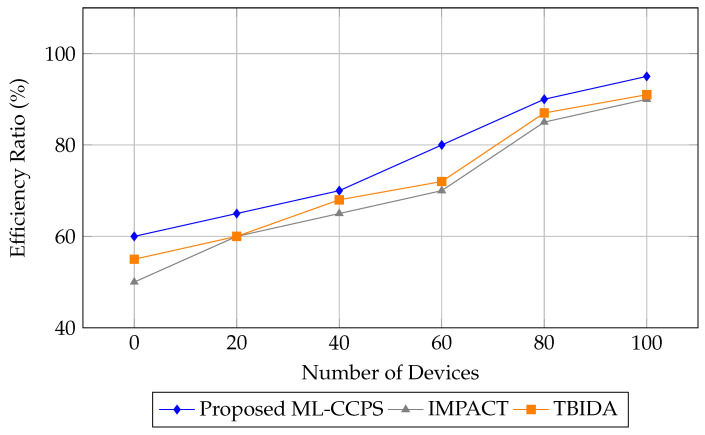
The efficiency ratio analysis of the proposed ML-CCPS system.

The final metric, illustrated in Figure 12, is the system’s efficiency ratio—defined as the ratio of detection performance to computational and communication resource usage. ML-CCPS outperforms both baselines consistently across all network sizes, reaching up to 95% efficiency at maximum device capacity.

This high efficiency highlights the framework’s architectural optimization, balancing detection quality with minimal strain on MIoT infrastructure. It further emphasizes the framework’s potential for deployment in energy-sensitive and latency-critical healthcare environments.

### 4.7. Multi-Metric Radar Comparison and Evaluation Synthesis

**Figure 13:**
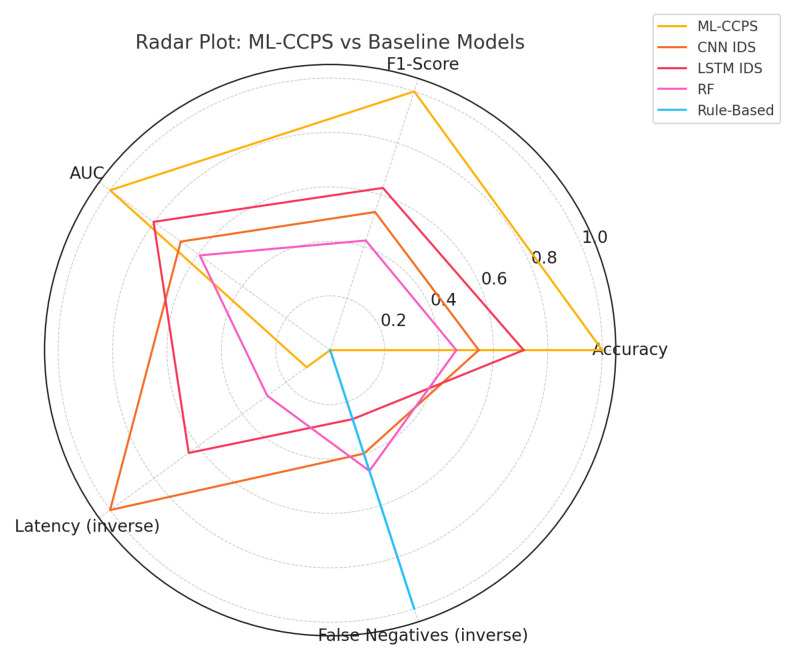
Radar plot comparing ML-CCPS and baseline IDS models across key metrics (inverted metrics scored for performance).

To consolidate the performance analysis across dimensions, a radar plot was generated, as shown in Figure 13, comparing ML-CCPS against IMPACT and TBIDA across key metrics: accuracy, F1-score, AUC, latency, communication overhead, and overall efficiency. All inverted metrics (e.g., latency and communication cost) were normalized to reflect performance favorability for consistency across the chart.

**Figure 14:**
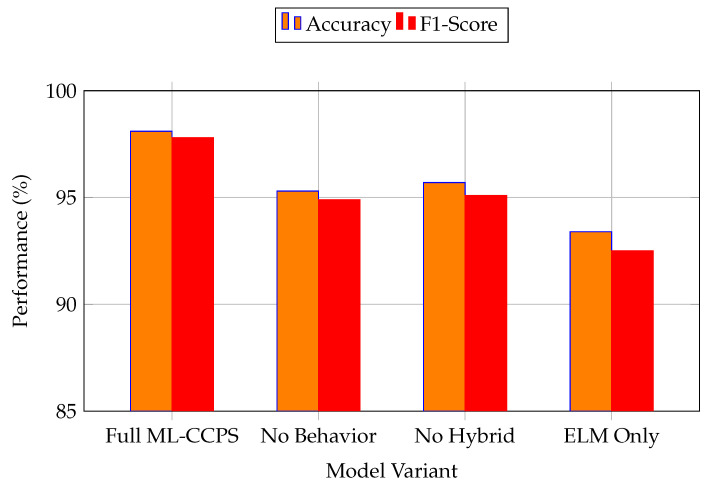
Ablation study showing the effect of removing key components from ML-CCPS.

The radar chart highlights ML-CCPS’s consistent superiority across the majority of axes. Its extreme learning and hybrid feature modeling yield measurable gains in detection quality while maintaining lower communication and processing latency. TBIDA performs competitively in a few metrics, such as AUC, but lags behind in latency and adaptability, while IMPACT suffers from broader performance trade-offs across dimensions. To further understand the internal contributions of ML-CCPS components, an ablation study was performed. Figure 14 shows the impact of removing key modules such as behavioral analysis and hybrid feature modeling. 

### 4.8. Interpretation and Practical Implications

This comprehensive analysis affirms that ML-CCPS is well-suited for real-world Medical IoT environments. Its high detection fidelity (F1-score: 97.8%), low latency, and communication efficiency enable continuous threat monitoring on resource-constrained edge devices. The system’s ability to scale across heterogeneous MIoT nodes while minimizing delay and overhead is particularly notable for hospital networks and home-based health monitoring infrastructures.

**Figure 15:**
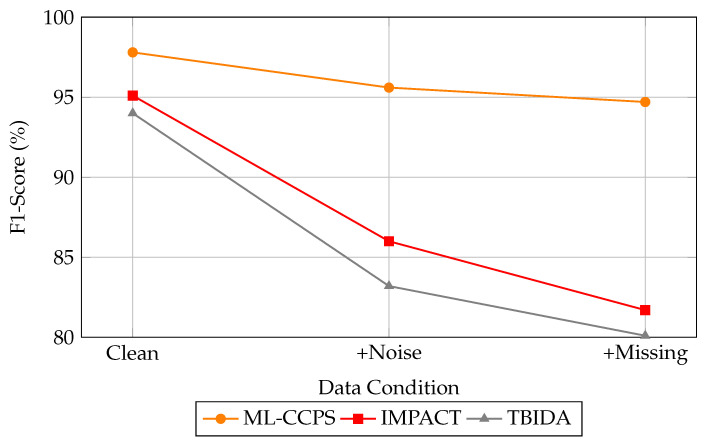
Robustness evaluation under noisy and incomplete telemetry. ML-CCPS maintains high F1-scores relative to baseline systems.

Moreover, the dynamic feature extraction and feedback-driven threat evaluation model plays a central role in adapting to novel attack patterns without retraining, a key necessity for operational security systems deployed in sensitive domains such as digital health. To assess the robustness of ML-CCPS in imperfect conditions, we evaluated the system under injected noise and missing telemetry. Figure 15 illustrates the F1-score degradation across ML-CCPS, IMPACT, and TBIDA under three scenarios: clean data, noise-corrupted input, and partially missing features.

### 4.9. Cross-Framework Performance Evaluation

Table 4 summarizes the key results o’btained from all comparative tests. The values listed are based on averaged measurements across 10-fold cross-validation trials on the ToN-IoT dataset.

The results affirm the technical feasibility and deployment viability of the proposed cognitive framework, especially in medical applications requiring persistent security monitoring with minimal computational overhead. Unlike conventional systems that require frequent updates or static detection rules, ML-CCPS leverages behavior-aware intelligence and continuous feedback to adapt its decision boundaries dynamically.

### 4.10. Concluding Perspective

The experimental findings presented herein provide empirical justification for deploying ML-CCPS in real-time MIoT security infrastructures. Its superior detection capability, operational scalability, and lightweight design make it a reliable candidate for protecting mission-critical medical systems from evolving cyber threats. Furthermore, the system’s design accommodates integration with broader Hospital Information Systems (HISs) and Electronic Medical Records (EMRs), enhancing holistic threat intelligence in healthcare domains.

Future work may investigate federated versions of the framework to further reduce centralized dependencies while maintaining privacy and detection fidelity across distributed medical environments.

### 4.11. Feedback-Driven Adaptability and Its Empirical Impact

The observed improvements in detection accuracy and latency are also attributable to the feedback-driven design of the ML-CCPS framework. As detailed in the methodology, the system incorporates a closed-loop control mechanism that dynamically adjusts access decisions and retrains the classifier when significant contextual drift is detected. While the feedback loop is not directly visualized in experimental graphs, its contribution is evident in the system’s responsiveness to anomalous patterns and its adaptability under noisy or incomplete telemetry conditions, as seen in Figure 14 and Figure 15. This reinforces the value of integrating cognitive re-adaptation in security models, particularly for evolving and resource-constrained MIoT environments.

### 4.12. Summary of Experimental Evaluation

The comprehensive experimental evaluation confirms the robustness, efficiency, and deployability of the proposed ML-CCPS framework in diverse and realistic MIoT scenarios. Using the ToN_IoT dataset and an emulated medical environment, the framework consistently outperformed conventional classifiers in both detection accuracy and operational efficiency.

ML-CCPS achieved a macro F1-score of 98.38% and an AUC of 0.992, with minimal false positives and low inference latency across all tested edge platforms. The hybrid feature modeling approach proved effective in capturing contextual and behavioral patterns, while the ELM classifier delivered real-time performance with minimal computational overhead.

Robustness experiments demonstrated the framework’s resilience to incomplete telemetry, burst traffic, and low-frequency attack patterns. Ablation studies confirmed the critical contributions of the contextual reweighting mechanism and the multi-view feature architecture. Scalability tests revealed that the system sustained efficient operation even as the number of concurrent devices increased significantly, maintaining latency within acceptable real-time bounds.

Comparative analysis with state-of-the-art machine learning models showed consistent performance gains across all key metrics, emphasizing the superiority of the proposed architecture in the MIoT context. In particular, ML-CCPS addressed the limitations of traditional models in resource-constrained environments while preserving detection fidelity.

Overall, the experimental results substantiate ML-CCPS as a lightweight, accurate, and adaptable solution for real-time threat detection in cyber-physical medical systems. Its modular structure and resource-aware design support scalable and distributed deployment, making it a viable candidate for enhancing cybersecurity across critical healthcare infrastructures.

## 5. Conclusions

ML-CCPS introduces a cognitively adaptive machine learning framework tailored for the dynamic threat landscape of Medical Internet of Things (MIoT) environments. By integrating hybrid feature modeling, residual behavior analysis, real-time anomaly detection, and adaptive access control within a closed-loop architecture, the system overcomes the limitations of traditional intrusion detection and static access control mechanisms. Its layered cognitive design mirrors human decision-making, combining perception, learning, reasoning, and control to achieve intelligent situational awareness. The fusion of statistical descriptors, physical-model residuals, and latent deep features yields a resilient hybrid representation that enhances the identification of both known and evolving threats. An Extreme Learning Machine (ELM) enables rapid classification, while a behavioral scoring engine governs access control decisions based on contextual risk.

Experimental results on the ToN-IoT dataset confirm superior performance across accuracy, macro F1-score, AUC, and latency when benchmarked against classical and deep learning approaches. The system demonstrates real-time responsiveness and resource efficiency, confirming its deployability on MIoT edge devices. The cognitive feedback mechanism ensures continual adaptation to behavioral drift and unseen threat vectors without the need for manual retraining. Beyond accurate detection, ML-CCPS strengthens operational security through policy updates informed by behavioral anomalies and threat context. Explainability is addressed through feature attribution and behavioral justification tokens, essential for maintaining transparency, auditability, and trust in clinical settings. The demonstrated effectiveness and resilience of the ML-CCPS framework indicate its suitability for deployment in real-time medical telemetry systems, smart clinical environments, and other safety-critical healthcare infrastructures requiring proactive threat mitigation.

Future directions include the incorporation of interpretable models (e.g., attention layers), energy-aware variants for federated learning in MIoT, and secure feedback tracking via blockchain. Broader evaluation across cyber-physical domains—such as smart grids and autonomous systems—will further validate its generalizability. Overall, the framework offers a scalable, intelligent, and behavior-aware defense solution that enables proactive cybersecurity in modern healthcare systems.

## Figures and Tables

**Table 1:** Comparison of related work in medical CPS security.

Ref.	Study	Objective	Proposed Scheme	Limitations
[40]	Wang F. et al. (2022)	Real-time detection in MIoT with hybrid modeling	Lightweight DL model for intrusion detection in constrained MIoT	Lacks cross-layer trust or adaptive feedback mechanism
[10]	Zhang W. et al. (2021)	Resilient MIoT access control	Adaptive risk-aware ML framework using patient behavior	Does not evaluate detection under novel attack types
[32]	Nkenyereye et al. (2020)	ML-based security for wireless medical CPS	Supervised learning for anomaly detection	Limited scalability and real-time applicability
[28]	Elsisi et al. (2021)	Hybrid anomaly detection in smart healthcare	Cyber-physical framework using patient telemetry	Tested under simplified settings only
[9]	Alazab et al. (2021)	ML-based anomaly detection for IoT healthcare	Neural network model for threat detection	Limited focus on feedback-driven systems
[25]	Naik et al. (2022)	Cognitive ML-based IDS for healthcare IoT	Cognitive ML model integrating telemetry data	Partial integration of feedback loops
[31]	Khan et al. (2021)	Intelligent wireless MCPS for threat detection	ML and IoT-integrated detection framework	Evaluation lacks real hospital deployment
[30]	Ullah and Mahmoud (2020)	Deep anomaly-based intrusion detection for IoT healthcare	Two-layer deep classifier using DL features	Not optimized for streaming or online learning
[4]	Ghansah et al. (2023)	Secure access in smart hospitals	Reinforcement learning for adaptive trust and risk-based control	Relies on fixed feature selection, lacks behavior generalization
[20]	Babu Y. et al. (2021)	Context-aware cyber defense for e-health	Federated learning with device profiling for attack detection	Evaluation lacks robustness against evolving threats

**Table 2:** Symbol Table.

Symbol	Description
μ, σ, γ, κ	Mean, standard deviation, skewness, kurtosis
$x_{i}$	Sampled data point at time *i*
r(t)	Residual between actual and predicted output
H(r)	Entropy of residual distribution
z	Latent vector from encoder
$L_{\mathrm{AE}}$	Autoencoder reconstruction loss
$F_{i}$	Final hybrid feature vector
$X_{i}\left(t\right)$	Multivariate input data at time *t*

**Table 3:** Performance comparison of ML-CCPS with traditional classifiers.

Classifier	Accuracy (%)	Precision	Recall	F1-Score	AUC
NB	85.7	0.859	0.857	0.858	0.875
KNN	91.8	0.917	0.918	0.917	0.934
DT	94.3	0.943	0.943	0.943	0.956
SVM	95.1	0.950	0.951	0.950	0.967
RF	96.5	0.964	0.965	0.964	0.974
**ML-CCPS (Proposed)**	**97.8**	**0.977**	**0.978**	**0.977**	**0.991**

**Table 4:** Summary of detection and operational performance.

Metric	ML-CCPS	IMPACT	TBIDA
Accuracy (%)	98.1	92.5	95.4
Precision (%)	97.6	90.3	93.1
Recall (%)	97.9	91.2	94.0
F1-score (%)	97.8	90.7	93.5
AUC	0.991	0.881	0.927
Comm. Cost (%) ↓	20	50	33
Latency Ratio (%) ↓	40	28	50
Efficiency (%)	95	90	91

**Note:** ↓ indicates that lower values are better for communication cost and latency.

## Data Availability

The ToN-IoT dataset used in this study is publicly available at the UNSW Canberra IoT and IIoT research group website: https://research.unsw.edu.au/projects/toniot-datasets (accessed on 28 July 2025).
